# Study on Additional Carrier Sensing for IEEE 802.15.4 Wireless Sensor Networks

**DOI:** 10.3390/s100706275

**Published:** 2010-06-24

**Authors:** Bih-Hwang Lee, Ruei-Lung Lai, Huai-Kuei Wu, Chi-Ming Wong

**Affiliations:** 1 Department of Electrical Engineering, National Taiwan University of Science and Technology, 43 Keelung Rd., Section 4, Taipei 106, Taiwan; E-Mails: tony@mail.vnu.edu.tw (R.-L.L.); chiming@just.edu.tw (C.-M.W.); 2 Department of Information Networking and System Administration, Ling Tung University, 1 Ling tung Rd., Taichung 408, Taiwan; E-Mail: hkwu@ccg.ee.ntust.edu.tw (H.-K.W.)

**Keywords:** wireless sensor network, IEEE 802.15.4, carrier sense multiple access with collision avoidance (CSMA/CA), medium access control (MAC)

## Abstract

Wireless sensor networks based on the IEEE 802.15.4 standard are able to achieve low-power transmissions in the guise of low-rate and short-distance wireless personal area networks (WPANs). The slotted carrier sense multiple access with collision avoidance (CSMA/CA) is used for contention mechanism. Sensor nodes perform a backoff process as soon as the clear channel assessment (CCA) detects a busy channel. In doing so they may neglect the implicit information of the failed CCA detection and further cause the redundant sensing. The blind backoff process in the slotted CSMA/CA will cause lower channel utilization. This paper proposes an additional carrier sensing (ACS) algorithm based on IEEE 802.15.4 to enhance the carrier sensing mechanism for the original slotted CSMA/CA. An analytical Markov chain model is developed to evaluate the performance of the ACS algorithm. Both analytical and simulation results show that the proposed algorithm performs better than IEEE 802.15.4, which in turn significantly improves throughput, average medium access control (MAC) delay and power consumption of CCA detection.

## 1. Introduction

The IEEE 802.15.4 standard [1] is uniquely designed to meet the requirements of low rate wireless personal area networks (LR-WPANs) to enable wireless sensor network applications [2–4]. Wireless sensor networks based on IEEE 802.15.4 are being increasingly deployed for different applications with the advances in micro-sensors, wireless networking and embedded processing technologies, and their applications include environmental monitoring, industrial sensing and diagnostics, health care and data collecting for battlefield awareness, *etc*. Generally speaking, IEEE 802.15.4 supports both star and peer-to-peer topologies. In star topology, a coordinator node is used to establish and maintain a WPAN. In peer-to-peer topology, a node can communicate with several nodes within its transmission range.

The IEEE 802.15.4 medium access control (MAC) protocol employs slotted carrier sense multiple access with collision avoidance (CSMA/CA) to access the channel and uses a random backoff algorithm to reduce the collision probability. To save power consumption in IEEE 802.15.4 networks, the slotted CSMA/CA uses a blind backoff process instead of a traditional backoff procedure. To transmit data, a node performs carrier sensing only when the backoff process is completed, and this causes lower channel utilization and longer average delays. To further improve the slotted CSMA/CA, a hybrid MAC protocol integrating CSMA and time division multiple access (TDMA) for wireless sensor networks has been proposed [5–7]. The enhanced backoff (EB) mechanism shifts the range of backoff period (BP) to reduce redundant backoffs and clear channel assessments (CCAs) [8], but the average delay also possibly increases. Therefore, we propose an additional carrier sensing (ACS) algorithm based on the IEEE 802.15.4 acknowledgement mode to detect the channel condition whenever the second CCA detects a busy channel. It can provide accurate information that the busy channel is caused by data or an acknowledged packet transmission in the second CCA detection. The transmission can then start after the acknowledged packet.

It does not seem so easy to find a suitable mathematical model to analyze IEEE 802.15.4 model. In [9,10] the authors develop an IEEE 802.15.4 analytical model having the same Markov formulation and assumptions as Bianchi for IEEE 802.11[15], but it fails to match simulation results. A Markov model developed by Park [11] is inaccurate, and an embedded Markov model proposed by Lee [12] is incomplete as well. Finally we develop a Markov chain model for ACS algorithm by integrating the models from [13,14]. By analysis and simulation experiments, ACS improves the throughput and delay performance but does not increase the energy consumption.

## 2. Description of ACS Algorithm

In IEEE 802.15.4 networks, each node communicates with the coordinator by using the slotted CSMA/CA in the contention access period (CAP). To transmit a packet, a node first delays a BP determined by randomly choosing from 0 to (2^BE^ − 1) slots, where BE is a backoff exponent and initially set to the value of *aMinBE*, and then performs the first CCA to detect channel condition. If the channel is idle in the first CCA detection, it will perform the second CCA to detect the channel again. If the results of both CCA channel detecting processes are idle, the node will start to transmit its data packet and waits for the acknowledged packet from the coordinator after finishing the data packet transmission, and the duration between the last transmission slot and the first acknowledment slot has been defined as t_ACK_, which will occupy one slot. In general, the acknowledged packet should occupy two slots. Conversely, if any CCA detects the channel being used, it will reassign a BP between 0 and (2^(BE + 1)^ − 1) slots for delay and attempt CCA again, where BE can be increased to the maximum value of *aMaxBE*. The transmission fails if the number of backoff attempts (NB) exceeds the value *macMaxCSMABackoffs*. In this paper, CCA1 and CCA2 will be used for the first CCA and second CCA, respectively.

To use the information in CCA1 and CCA2 efficiently, ACS enhances the performance in the IEEE 802.15.4 slotted CSMA/CA. Considering IEEE 802.15.4 in the beacon-enabled with acknowledged mode, CCA2 mainly fails in only two cases. First, there is at least one of the other nodes successfully performing CCA2 at the same slot, when the target node performs CCA1. In this case, the CCA2 performed by the target node will detect a busy channel which is caused by a node starting to transmit its packet in the same slot. The second possible case is that the target node performs CCA1 while at least one of the other nodes waits for the acknowledged packet after finishing transmission. The target node will detect an idle channel in CCA1 for the duration of the t_ACK_ slot. If the node successfully transmits, the CCA2 performed by the target node will detect a busy channel because the coordinator is replying to the acknowledged packet in the same slot. Clearly, in the second possible case of CCA2 failure, we can make sure that the target node can transmit its data packet after the next CCA2 failed slot. Therefore, ACS will perform the third CCA (also called CCA3) to detect the channel after the next CCA2 failed slot. CCA2 absolutely fails in the first case and the transmission is not allowed if CCA3 detects a busy channel. CCA2 fails in the second case but the data packet can be transmitted right after CCA3, if CCA3 detects an idle channel.

**Figure 1.**
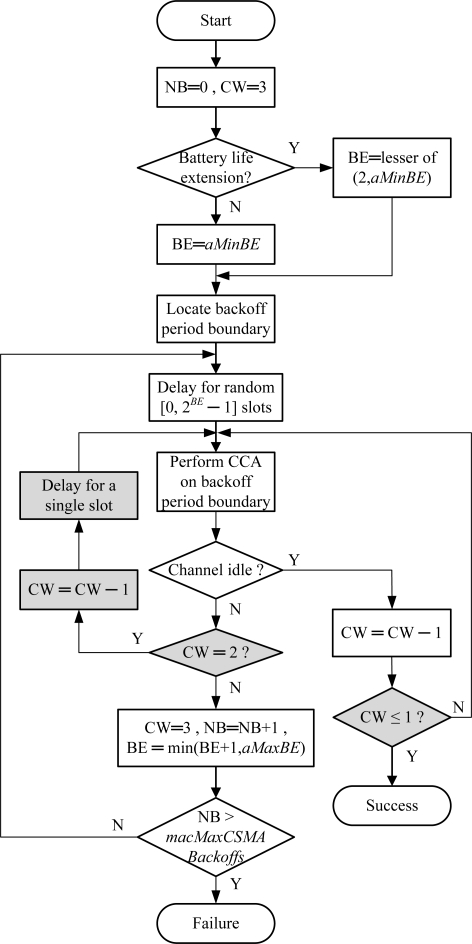
The flowchart of the ACS algorithm.

Basically, the flowchart of the ACS algorithm is obtained by slightly modifying the IEEE 802.15.4 standard, as shown in Figure 1. We first set the contention window (CW) to 3 for three CCAs and add a conditional decision for checking the result of CCA2 detection. If CW is equal to 2 after detecting a busy channel, it must be that CCA2 failed. We perform CCA3 after delaying a single slot to see if CCA2 fails in the first case or the second case and further decide to transmit or go to the next backoff stage.

## 3. Analysis of the ACS Algorithm

In this section, we analyze the proposed ACS algorithm based on the IEEE 802.15.4 slotted CSMA/CA in the case of acknowledged uplink data transmission with unsaturated traffic conditions. We consider a single hop with star topology consisting of a coordinator and N sensor nodes under the assumptions of ideal channel conditions without hidden nodes and capture effects. We assume that data packets arrive at each sensor node according to the Poisson process with rate λ for uplink transmission. According to the IEEE 802.15.4 specification, an aUnitBackoffPeriod (UBP) contains 20 symbols, and one symbol contains 4 bits, *i.e.*, a UBP contains 80 bits. We also assume that the length of data packet *L_d_* is fixed and occupies 12 UBPs for transmission, while t_ACK_ and the length of the acknowledged packet L_ACK_ occupy 1 and 2 UBPs, respectively. We derive the stationary probability that a node attempts the first carrier sensing in a random chosen UBP, and analyze throughput, MAC delay and the number of CCAs sent before transmission to understand the energy consumed by CCAs.

### 3.1. System Model and Throughput Analysis

**Figure 2.**
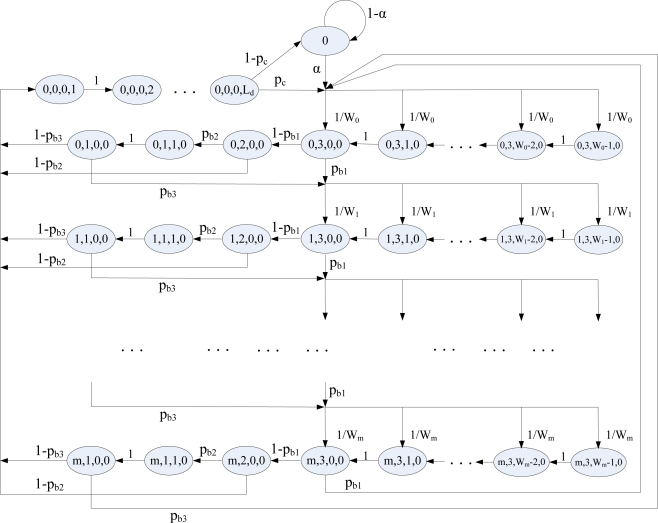
The Markov chain model for the ACS algorithm.

Let *s(t)*, *c(t)*, *b(t)* and *y(t)* be the stochastic processes representing the backoff stage, CW value, backoff counter and transmitting slot at the boundary of slot *t* for a given sensor node, respectively. We have *s(t)* ∈ {0,1,…,*m*} by assuming that *m* is the maximum backff stage and is equal to *aMaxBE − aMinBE*. Since CCA detection occurs three times in the ACS algorithm, we have *c(t)* ∈ {0,1,2,3}. The backoff counter in backoff stage *i* will be uniformly selected from [0, *W_i_* − 1] where *W_i_* = 2^(*aMinBE* + *i*)^ and 0 ≤ *i* ≤ *m*; then we have *b(t)* ∈ {0,1,…, *W_i_* − 1}. Since *L_d_* is the total transmission slots, we have *y(t)* ∈ {1,…, *L_d_*}, where the state *y(t)* = 0 represents nothing for transmitting or receiving. The set of processes {*s(t)*, *c(t)*, *b(t)*, *y(t)*} defines the state of a node at the boundaries of time slots. The behavior of a single device is described by the discrete-time Markov chain shown as Figure 2. This Markov chain model combines Jung’s model [13] with Pollin’s model [14] and further considers the CCA3 operation. In the Markov chain model, the state {0} represents the idle state whenever a node has no data packet for transmission. In Figure 2, p_c_ is the collision probability caused by at least one of N − 1 remaining nodes to senses successfully and transmits; p_b1_ is the probability of detecting a busy channel in CCA1, while p_b2_ and p_b3_ are probabilities that a node detects a busy channel before performing CCA2 and CCA3, respectively, given that the channel is idle by performing CCA1 and the previous CCA2 is failed, respectively. The term α represents the transition probability of data packet arrival as obtained by Equation (1) [13], where *T_slot_* is the duration of a time slot:

(1) $$\alpha =\int_{0}^{T_{\mathrm{slot}}}\lambda e^{-\lambda t}\mathrm{dt}$$

Let us denote P_K1_ and P_K2_ to be the probabilities of entering the next backoff stage and transmitting a data packet from a node in a certain backoff stage, respectively, where P_K1_ and P_K2_ can be further obtained by P_K1_ = p_b1_ + (1 − p_b1_)p_b2_p_b3_ and P_K2_ = (1 − p_b1_)[(1 − p_b2_) + p_b2_(1 − p_b3_)]. The other transition probabilities associated with the Markov chain are presented as follows.

Equation (2) states the probability that the backoff counter is decreased after each slot. Equation (3) gives the probability of finding a busy channel in CCA1 or CCA3 and a node uniformly selects a state in the next backoff stage. Equation (4) gives the probability to uniformly choose a state for starting a new transmission when the previous transmission is successful or retransmission attempt after the previous transmission failure or reaches to the maximum backoff stage. Equation (5) states the probability of the decreased backoff counter to perform CCA3. Equation (6) states the probability of transmission after the backoff counter reaches zero. Equation (7) states the probability of transmitting in the next slot.

(2) $$P\{i,3,k-1,0|i,3,k,0\}=1,\begin{array}{cc} & i\in [0,m],\begin{array}{cc} & k\in [0,W_{i}-1] \end{array} \end{array}$$

(3) $$P\{i,3,k,0|i-1,3,0,0\}=\frac{p_{b1}+(1-p_{b1})p_{b2}p_{b3}}{W_{i}}=\frac{P_{K1}}{W_{i}},\;i\in [0,m],\;k\in [0,W_{i}-1]$$

(4) $$\begin{array}{c} P\{0,3,k,0|m,3,0,0\}=\frac{P_{K1}+(1-p_{b1})[(1-p_{b2})+p_{b2}(1-p_{b3})][p_{c}+\alpha (1-p_{c})]}{W_{0}}\\ \begin{array}{cc} =\frac{P_{K1}+P_{K2}[p_{c}+\alpha (1-p_{c})]}{W_{0}}, & k\in [0,W_{0}-1] \end{array} \end{array}$$

(5) $$P\{i,1,0,0|i,1,1,0\}=1,\begin{array}{cc} & i\in [0,m] \end{array}$$

(6) $$P\{0,0,0,1|i,3,0,0\}=P_{K2},\;i\in [0,m]$$

(7) $$P\{0,0,0,l+1|0,0,0,l\}=1,\;l\in [1,L_{d}-1]$$

The closed-form solution of the Markov chain can be obtained by Equations (2)–(7) with the chain regularities as follows. Let *b_i,j,k,l_* be the stationary distribution of the Markov chain, *i.e.*,

$$b_{i,j,k,l}=\underset{t\to \infty }{\text{lim}}P\{s(t)=i,c(t)=j,b(t)=k,y(t)=l\}$$

for *i* ∈ [0,*m*], *j* ∈ [0,3], *k* ∈ [0,*W_i_* − 1], *l* ∈ [0,*L_d_*], where *b*_0_ = *P*{0}. By using Equation (3), we can obtain b*_i,3,0,0_* shown as Equation (8). The steady-state probabilities to perform CCA2 and CCA3 can be obtained by equations (9) and (10), respectively. *b_0,0,0,1_* and *b_0_* can also be obtained by equations (11) and (12), respectively. Consequently, *b_0,3,k,0_* and *b_i,3,k,0_* can be expressed by equations (13) and (14), respectively.

(8) $$b_{i,3,0,0}=P_{K1}b_{i-1,3,0,0}=P_{K1}^{i}b_{0,3,0,0}\;\;\;i\in [0,m]$$

(9) $$b_{i,2,0,0}=(1-p_{b1})b_{i,3,0,0}=(1-p_{b1})P_{K1}^{i}b_{0,3,0,0}$$

(10) $$b_{i,1,0,0}=b_{i,1,1,0}=p_{b2}b_{i,2,0,0}=p_{b2}(1-p_{b1})P_{K1}^{i}b_{0,3,0,0}$$

(11) $$b_{0,0,0,1}=P_{K2}\sum_{i=0}^{m}b_{i,3,0,0}=P_{K2}\sum_{i=0}^{m}P_{K1}^{i}b_{0,3,0,0}=\frac{P_{K2}(1-P_{K1}^{m+1})}{1-P_{K1}}b_{0,3,0,0}=(1-P_{K1}^{m+1})b_{0,3,0,0}$$

(12) $$b_{0}=\frac{\left(1-p_{c}\right)}{\alpha }b_{0,0,0,1}=\frac{\left(1-p_{c})(1-P_{K1}^{m+1}\right)}{\alpha }b_{0,3,0,0}$$

(13) $$b_{0,3,k,0}=\frac{W_{0}-k}{W_{0}}[\alpha b_{0}+p_{c}b_{0,0,0,1}+P_{K1}b_{m,3,0,0}]=\frac{W_{0}-k}{W_{0}}b_{0,3,0,0}\;k\;\in \;[0,W_{0}-1]$$

(14) $$b_{i,3,k,0}=\frac{W_{i}-k}{W_{i}}P_{K1}b_{i-1,3,0,0}=\frac{W_{i}-k}{W_{i}}P_{K1}^{i}b_{0,3,0,0}\;i\;\in \;[0,m],\;k\;\in \;[0,W_{i}-1]$$

Since the sum of probabilities in the Markov chain must be equal to one, we have:

(15) $$\sum_{i=0}^{m}\sum_{k=0}^{W_{i}-1}b_{i,3,k,0}+\sum_{i=0}^{m}b_{i,2,0,0}+\sum_{i=0}^{m}b_{i,1,0,0}+\sum_{i=1}^{L_{d}}b_{0,0,0,l}+\sum_{i=0}^{m}b_{i,1,1,0}+b_{0}=1$$

Therefore, *b_0,3,0,0_* can be obtained by the expression of *p_b1_*, *p_b2_*, *p_b3_* and *p_c_* as shown in Equation (16):

(16) $$b_{0,3,0,0}=\frac{1}{\frac{1}{2}\sum_{i=0}^{m}P_{K1}^{i}(1+W_{i})+\frac{1-P_{K1}^{m+1}}{1-P_{K1}}[(1-p_{b1})+2p_{b2}(1-p_{b1})]+(1-P_{K1}^{m+1})[L_{d}+\frac{\left(1-p_{c}\right)}{\alpha }]}$$

Let *φ* be the probability that a node performs CCA1 in a random chosen time slot when the backoff counter reaches zero without considering the backoff stage and independent across nodes, where *φ* can be expressed as Equation (17):

(17) $$\phi =\sum_{i=0}^{m}b_{i,3,0,0}=\sum_{i=0}^{m}P_{K1}^{m}b_{0,3,0,0}=\frac{1-P_{K1}^{m+1}}{1-P_{K1}}b_{0,3,0,0}$$

Accordingly, let *P_t_* be the transmission probability that at least one node senses the channel successfully, while *P_s_* is the successful transmission probability that a node senses the channel successfully and the others are not, which can be expressed by equations (18) and (19), respectively:

(18) $$P_{t}=P_{K2}(1-{\left(1-\varphi \right)}^{N})$$

(19) $$P_{s}=P_{K2}N\varphi {\left(1-\varphi \right)}^{N-1}$$

Let *P_coll_* be the collision probability of the entire system that can be obtained and shown as Equation (20):

(20) $$P_{\mathrm{coll}}=1-\frac{P_{s}}{P_{t}}=\frac{1-{\left(1-\varphi \right)}^{N}-N\varphi {\left(1-\varphi \right)}^{N-1}}{1-{\left(1-\varphi \right)}^{N}}$$

The closed forms of *p_b1_*, *p_b2_*, *p_b3_* and *p_c_* can be obtained by solving equations (17)–(20). *p_b1_* is the probability that a performing CCA1 node detects a busy channel caused by at least one of N-1 remaining nodes transmitting data packets or receiving ACK packets, shown as Equation (21) [14]:

(21) $$p_{b1}=P_{K2}(1-{\left(1-\varphi \right)}^{N-1})(L_{d}+L_{\mathrm{ACK}}(1-P_{\mathrm{coll}}))$$

**Figure 3.**
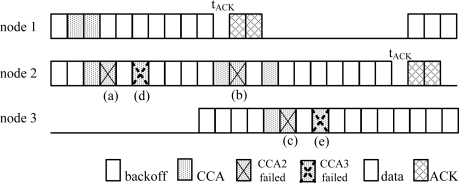
An example of ACS algorithm for three nodes.

The formula of *p_b2_* in the standard edition of IEEE 802.15.4 consists of two terms derived by the unacknowledged and acknowledged modes corresponding to slots (a) and (b) in Figure 3, respectively [14].

In the ACS algorithm, we still need to consider the other conditions that cause CCA2 to fail, that is, at least one of N-1 nodes successfully detects an idle channel in CCA3 whenever the target node is performing CCA1 at the same time. Consequently, the node performing successful CCA3 will start transmission in the next slot and further causes the target node to detect a busy channel in CCA2. In Figure 3, we know that CCA2 failed not only at slots (a) and (b), but also at slot (c). Besides, the probability of CCA2 failure occurring at slot (c) is the same as at slot (b). Thus, *p_b2_* can be obtained by Equation (22). Moreover, both events in the slots (d) and (e) are the only two cases that cause CCA3 failure. Clearly, the events in the slots (d) and (e) only follow the events in the slots (a) and (c), respectively, which means *p_b3_* is the combination of the events in both slots (a) and (c) and can be obtained by Equation (23). Finally *p_b1_*, *p_b2_*, *p_b3_* and *p_c_* can be obtained by solving the above four non-linear equations (21)–(24). Furthermore throughput *S* can be simply expressed by Equation (25) if *B* is denoted to be the bandwidth of the channel:

(22) $$p_{b2}=\frac{3-2P_{\mathrm{coll}}}{3-2P_{\mathrm{coll}}+\frac{1}{1-{\left(1-\varphi \right)}^{N}}}$$

(23) $$p_{b3}=\frac{2-P_{\mathrm{coll}}}{3-2P_{\mathrm{coll}}+\frac{1}{1-{\left(1-\varphi \right)}^{N}}}$$

(24) $$p_{c}=P_{K2}(1-{\left(1-\varphi \right)}^{N-1})$$

(25) $$S=L_{d}P_{s}B$$

### 3.2. Analysis of Average MAC Delay

In this subsection, we analyze the average MAC delay for each node. The MAC delay is defined as the time between packet arrival and transmission, so it can be obtained by simply counting the average number of states that a node experiences in the Markov chain. The average number of states also implies how many slots on average are experienced for a transmission whenever a new packet arrives.

Let *d_i_* be the average backoff counter in backoff stage *i* and also be the average number of states (slots) experienced by a node to perform backoff countdown procedure in backoff stage *i*, where *d_i_ = W_i_/2*. In Figure 2, a node may have two different paths, e.g., path1 and path2, to enter the next backoff stage with probabilities *P_C1_* and *P_C2_*, respectively, whenever it senses a busy channel in the current backoff stage, where *P_C1_ = p_b1_*, *P_C2_ = (1 − p_b1_)p_b2_p_b3_*. Therefore, a new arrival packet has in total *2^i^* different paths to enter the *i*^th^ backoff stage. Moreover, a node entering the next backoff stage by path2 will experience 3 more states than by path1, *i.e.*, it causes it to perform CCA3 three more times. Similarly, a node may have two different paths, e.g., path3 and path4, to successfully sense the channel and transmit in a certain backoff stage with probabilities *P_C3_* and *P_C4_*, respectively, where *P_C3_ = (1 − p_b1_)(1 − p_b2_)*, and *P_C4_ = (1 − p_b1_)p_b2_(1 − p_b3_)*. Therefore, a new arrival packet will have 2^*i* + 1^ different paths to successfully sense the channel and transmit in the *i*^th^ backoff stage. Furthermore, a node which senses the channel successfully and transmits by path4 will experience two more states than by path3, *i.e.*, it causes CCA3 to be performed two more times.

Let *D_i_* be the average number of states that a node experiences to successfully sense the channel and transmit in backoff stage *i*, and *D_1_* can be obtained by Equation (26):

(26) $$\begin{array}{c} D_{1}=(d_{0}+d_{1}+1)P_{C1}^{1}P_{C2}^{0}P_{C3}^{1}P_{C4}^{0}+(d_{0}+d_{1}+3)P_{C1}^{1}P_{C2}^{0}P_{C3}^{0}P_{C4}^{1}\\ +(d_{0}+3+d_{1}+1)P_{C1}^{0}P_{C2}^{1}P_{C3}^{1}P_{C4}^{0}+(d_{0}+3+d_{1}+3)P_{C1}^{0}P_{C2}^{1}P_{C3}^{0}P_{C4}^{1} \end{array}$$

In the right hand side of Equation (26), the first two terms represent that a node fails to sense the channel in backoff stage 0 and enters backoff stage 1 with probability *P_C1_*, then it experiences *d_0_ + d_1_ + 1* or *d_0_ + d_1_ + 3* states for successfully sensing the channel and transmission with probability *P_C3_* or *P_C4_*, respectively. Furthermore, as mentioned above, the average number of states via path1 and path4 is *d_0_ + d_1_ + 3*, which includes two more states to perform a CCA3 than that via path1 and path3, *i.e.*, *d_0_ + d_1_ + 1*. Similarly, the last two terms represent a node entering backoff stage 1 with probability *P_C2_* and experiencing *d_0_+3+d_1_+1* or *d_0_+3+d_1_+3* states for successfully sensing the channel and transmission with probability *P_C3_* or *P_C4_*, respectively. The average number of states via path2 and path4 includes two more states to perform a CCA3 than that via path2 and path3. Summarily, *D_i_* can be obtained by Equation (27), where the exponents of *q* and *r* are the remainder of *j/2* and *(j + 1)/2*, respectively, while *v* and *u* are the number of paths via path1 and path2, respectively. Since path1 and path2 are mutually exclusive, there are totally *2^i^* different paths to enter the *i*^th^ backoff stage for a new arrival packet as the mentioned above. The term *u* represents the number of paths via path2, which can be obtained by counting the number of 1s in the binary format of ⌊*j*/2⌋, and *v* is equal to *i*−*u.* Let *D_s_* be the summation of *D_i_* for *i* ∈ [0,*m*] shown as Equation (28), where *X_j_* is a sequence representing the number of states caused by different possible paths to transmit in the *i*^th^ backoff stage shown as Equation (29) for *j* ∈[0,2*^i^*^+1^−1]. Finally, the average MAC delay *D_av_* can be obtained by Equation (30).

(27) $$D_{i}=\sum_{j=0}^{2^{i+1}-1}\left[\left(\sum_{k=0}^{i}d_{k}+X_{j}\right)P_{C1}^{v}P_{C2}^{u}P_{C3}^{r}P_{C4}^{q}\right]$$

(28) $$D_{s}=\sum_{i=0}^{m}D_{i}$$

(29) $$X_{j}=\{\begin{array}{ll} 1, & j=0\\ 3, & j=1\\ X_{k}+3, & j=2^{i}+k,\;\;0\leq k<2^{i+1}-2^{i},\;\;i=1,2,... \end{array}$$

(30) $$D_{\mathrm{av}}=T_{\mathrm{slot}}D_{s}\sum_{i=0}^{\infty }{\left(P_{C1}+P_{C2}\right)}^{i}=\frac{T_{\mathrm{slot}}D_{s}}{1-(P_{C1}+P_{C2})}$$

To consider if the additional CCA3 consumes more power, we can simply count the average number of CCAs sent by each node between packet arrival and transmission to see the power consumption caused by all sent CCAs. Clearly, it is very similar to obtaining the average MAC delay; the number of CCAs can be obtained and shown as Equation (31) by using the backoff stage instead of the average backoff counter in Equation (27), where *Y_j_* is a little different from *X_j_* in Equation (29) and shown as Equation (32) if we only consider the average number of CCAs and neglect the single slot backoff in CCA3. Therefore, the average number of CCAs sent before transmission, *N_av_CCA_*, is given by Equation (33):

(31) $$N_{\mathrm{CCA}}=\sum_{i=0}^{m}\sum_{j=0}^{2^{i+1}-1}\left[\left(i+1+Y_{j}\right)P_{C1}^{v}P_{C2}^{u}P_{C3}^{r}P_{C4}^{q}\right]$$

(32) $$Y_{j}=\{\begin{array}{ll} 1, & j=0\\ 2, & j=1\\ Y_{k}+2, & j=2^{i}+k,\;0\leq k<2^{i+1}-2^{i},\;\;i=1,2,...\; \end{array}$$

(33) $$N_{\mathrm{av}\_\mathrm{CCA}}=N_{\mathrm{CCA}}\sum_{i=0}^{\infty }{\left(P_{C1}+P_{C2}\right)}^{i}=\frac{N_{\mathrm{CCA}}}{1-(P_{C1}+P_{C2})}$$

## 4. Simulation Experiments

In this section, a simulation experiment is performed using the Visual C++ program. We consider a single hop with star topology consisting of a coordinator and *N* sensor nodes assuming ideal channel conditions. All nodes can communicate with the coordinator at the full data rate of 250 kbps and no capture effect is considered. We assume that the inactive period and CFP can be neglected. The packet length *L_d_* is fixed at 120 bytes. The packet arrival rate of each node follows the Poisson distribution with a mean of *λ*, that is, the traffic load is equal to (*N* × *λ* × *L_d_* × 8)/250 kbps. We further compare with the IEEE 802.15.4 standard for throughput, average MAC delay and the number of CCAs sent before packet transmission, where the analytical results are obtained from [14]. Table 1 summarizes the system parameters used for simulation.

**Figure 4.**
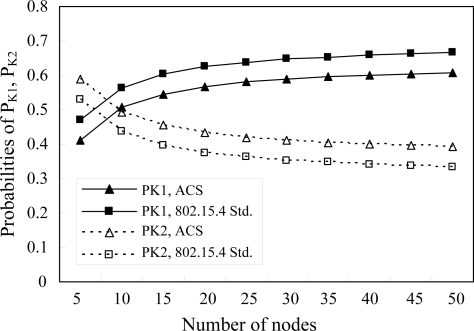
The probabilities of P_K1_ and P_K2_ *versus* number of nodes with traffic load = 0.6.

Figure 4 shows the probabilities of *P_K1_* and *P_K2_ versus* the number of nodes for the proposed ACS algorithm and the IEEE 802.15.4 standard, respectively. It shows that ACS has less *P_K1_* and greater *P_K2_* than IEEE 802.15.4, *i.e.*, a node has less probability to enter the next backoff stage and greater probability to transmit a data packet in a certain backoff stage by using ACS. Therefore, ACS should have better throughput and MAC delay performance than IEEE 802.15.4.

**Figure 5.**
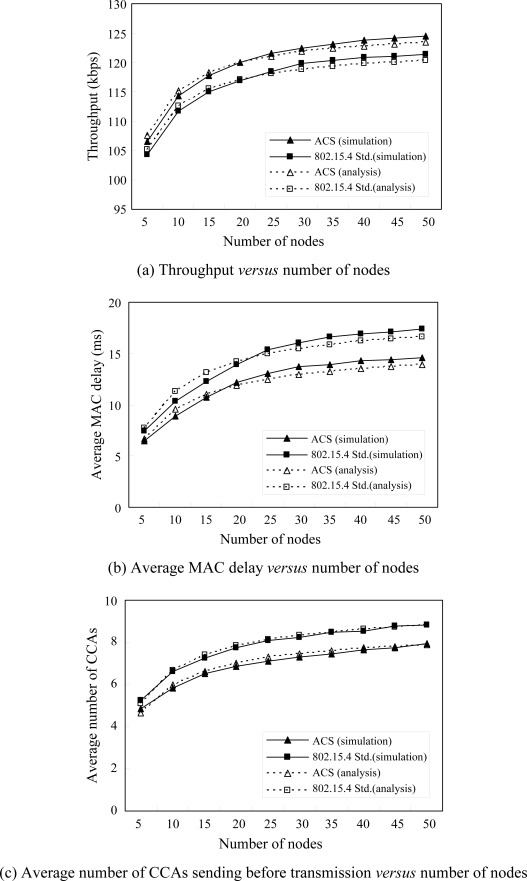
Throughput, average MAC delay and number of CCAs sent before transmission *versus* the number of nodes with traffic load = 0.6 by analytical and simulation.

Figure 5 shows the results of the performance measurements *versus* the number of nodes while traffic load is equal to 0.6. As expected by *P_K1_* and *P_K2_* as shown in Figure 4, Figures 5(a) and 5(b) show that ACS has greater throughput and smaller average MAC delay than IEEE 802.15.4. In Figure 5(c), we compare the number of CCAs sent before transmission to see the power consumed by CCAs. The results show that ACS has the smaller number of CCAs than IEEE 802.15.4, which means that ACS uses less power consumed by CCAs than IEEE 802.15.4.

**Figure 6.**
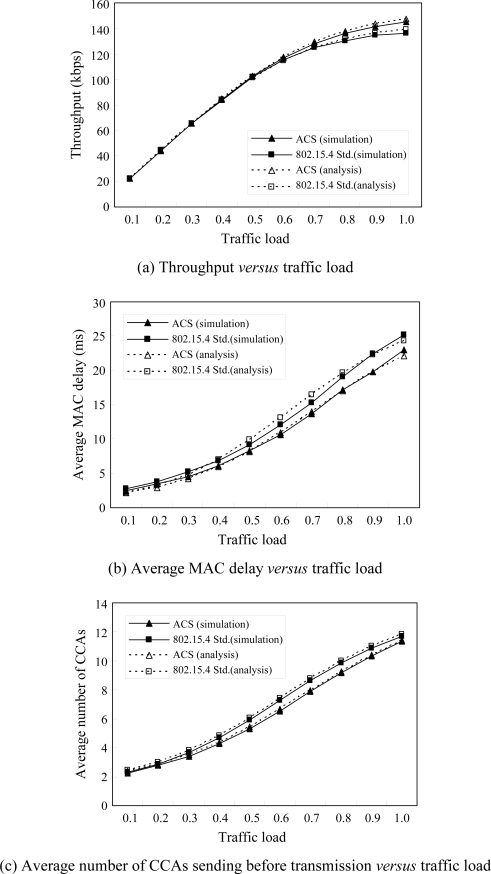
Throughput, average MAC delay and number of CCAs sent before transmission *versus* traffic load with the number of nodes = 15 by analytical and simulation.

Figure 6 shows the results of the performance measurements *versus* the traffic load while the number of nodes is equal to 15. It is obvious that the results of throughput, average MAC delay and power consumed by CCAs are almost the same for both ACS and IEEE 802.15.4 as the traffic load is light; but ACS performs better than IEEE 802.15.4 when the traffic load gradually increases. It is obvious that ACS alleviates the collision probability under the heavy traffic load.

## 5. Conclusions

In this paper, the ACS algorithm based on the IEEE 802.15.4 beacon-enabled with acknowledgement mode is proposed. It uses an additional CCA (*i.e.*, CCA3) to see if a data packet can be transmitted after the preceeding acknowledged packet or not. In doing so it seems to increase the number of CCAs used to detect the channel status for a transmission; conversely, it saves the number of CCAs detections for the future backoff stage if CCA3 is successful. The results obtained by the analytical model and simulation experiments show that the ACS algorithm significantly improves throughput, average MAC delay and power consumption of CCA detection, respectively. However, it is more complicated to solve for the multi-hop cluster topology than the single hop with star topology in IEEE 802.15.4 networks. Therefore, the ACS algorithm will be further expected to consider the hidden nodes problem in the multi-hop cluster topology and to improve the performance of the guaranteed time slot (GTS) allocation.

## Figures and Tables

**Table 1.** The system parameters for simulation.

Channel bandwidth	250 kbps
aUnitBackoffPeriod (UBP)	80 bits
MAC Header	2 UBP
Data payload	12 UBP
*t_ACK_*	1 UBP
*L_ACK_*	2 UBP
*aMinBE*	3
*aMaxBE*	5
*macMaxCSMABackoffs*	4
